# Increasing physical activity and decreasing sedentary activity in adolescent girls – The Incorporating More Physical Activity and Calcium in Teens (IMPACT) study

**DOI:** 10.1186/1479-5868-5-42

**Published:** 2008-08-21

**Authors:** Desiree Jones, Deanna M Hoelscher, Steven H Kelder, Albert Hergenroeder, Shreela V Sharma

**Affiliations:** 1 The Michael and Susan Dell Center for Advancement of Healthy Living, University of Texas Health Science Center, School of Public Health, Houston, Texas, USA; 2 The Michael and Susan Dell Center for Advancement of Healthy Living, University of Texas School of Public Health, Austin, Texas, USA; 3 Baylor College of Medicine, Adolescent Medicine Service and Sports Medicine Clinic, Texas Children's Hospital, Houston, Texas, USA

## Abstract

**Background:**

Lack of regular physical activity and consequent sub-optimal bone mass acquisition in youth has been implicated as a primary cause of adult-onset osteoporosis. IMPACT was a behavioral theory-based 1 1/2 year randomized controlled field study aimed at increasing bone accretion in middle school girls. The objective of this study was to determine the intervention effects of the IMPACT program upon key physical and sedentary activity endpoints among schools that participated in the IMPACT study. Endpoints examined included weight bearing physical activity (WBPA); moderate to vigorous physical activity (MVPA); vigorous physical activity (VPA); MET (metabolic equivalent) – weighted WBPA and MVPA; sedentary activity; before/after-school physical activity; and weekend physical activity.

**Methods:**

Primary data analysis using a pretest-posttest control group design was conducted utilizing mixed model analysis of covariance. Data gathered from the IMPACT cohort from 2000–2002 were analyzed to determine baseline versus follow-up differences in activity endpoints. Confounders investigated included ethnicity, body mass index, menarcheal status, participation in 7^th ^grade PE/athletics, friend/familial support and neighborhood safety.

**Results:**

Follow-up means were higher for participating intervention schools relative to control schools for all physical activity variables but were statistically significant only for the following variables: daily minutes of vigorous physical activity (mean difference between Intervention (I) and Control (C) = 6.00↑ minutes, 95% CI = 5.82–6.18, p = 0.05), daily after school activity minutes (mean difference between I and C = 8.95↑ minutes, 95% CI = 8.69–9.21, p = 0.04), and daily weekend activity minutes (mean difference between I and C = 19.00↑ minutes, 95% CI = 18.40–19.60, p = 0.05). The intervention significantly reduced duration of student daily TV/Video watching (mean difference between I and C = 12.11↓ minutes, 95% CI = 11.74–12.48, p = 0.05) and total daily sedentary activity minutes (mean difference between I and C = 16.99↓ minutes, 95% CI = 16.49–17.50, p = 0.04).

**Conclusion:**

A well designed and implemented school based health and physical activity intervention can result in a positive influence upon increasing physical activity levels and decreasing sedentary activity. Future interventions should consider a more structured intervention component to obtain significant changes in WBPA.

## Background

Osteoporosis is a disorder marked by micro-architectural deterioration of the bone resulting in bone fragility and an increased susceptibility to fractures [1-4]. For diagnostic purposes, the World Health Organization has defined osteoporosis as a bone mineral density value more than 2.5 standard deviations below the mean for normal young White women [4]. Based on this definition, an estimated 10 million individuals over age 50 in the United States presently have osteoporosis and an additional 33.6 million individuals over age 50 have low bone mass or "osteopenia" of the hip. These individuals are at risk for osteoporosis and its potential complications later in life [5-7]. Although the onset and manifestation of bone disease and osteoporosis occurs primarily in the elderly, it is now well established that the foundations and origins of imbalances in bone metabolism that eventually lead to overt disease are established in youth [8,9]. It has been suggested that one of the primary means of preventing osteoporosis is to affect modifying factors (such as diet and physical activity) that influence bone density so that peak bone mass is achieved during the first twenty years of life [2,8,10].

As a result of the large body of literature clearly establishing the benefits of physical activity in children and adolescents to overall health, and bone health in particular, international guidelines for adolescent physical activity have been established [11,12]. In spite of these recommendations, a recent national survey report by the Centers for Disease Control and Prevention has revealed that only 67% of high school students in the United States met the national recommendations for both moderate and vigorous physical activity in 2003 [13]. Since the 1980's, there have been several behavioral-theory based interventions in schools and other settings that have been implemented with the objective of increasing physical activity or improving biological endpoints such as bone mineral density through increased physical activity levels in youth [14-25]. The majority of these studies have not been formally evaluated with respect to the actual effectiveness of their physical activity programs in terms of average daily or weekly increases in duration or intensity of physical activity levels or evaluated effects on sedentary activities. Few of these interventions have been successful in increasing physical activity and/or decreasing sedentary activity in middle school-age girls [16,18,19]

IMPACT (Incorporating More Physical Activity and Calcium in Teens) was a multi-component intervention to promote bone health in middle school girls, with physical activity as one of the major intervention components. The purpose of this paper is to briefly describe the physical activity component of IMPACT and to report major results with respect to changes obtained in levels of weight-bearing physical activity (WBPA), moderate to vigorous physical activity (MVPA), vigorous physical activity (VPA), sedentary activities, before/after-school activities and weekend activities among students from schools that participated in the IMPACT intervention.

## Methods

### Study design

Twelve middle schools in Central Texas (six intervention and six control schools) were recruited and pair-matched based on school characteristics at baseline. The matching criteria used were student ethnicity, percent of students who were economically disadvantaged and percent girls in athletics. The study design was a randomized clinical trial with one school from each matched pair randomly assigned via computerized randomization to either the IMPACT program, or the control group (usual health program). Girls from the sixth grade who were enrolled in two semesters of physical education were eligible to participate in the study. At the beginning of the study, baseline measurements were conducted in the Fall of 2000, followed by interim measurements conducted in Spring 2001, and final measurements taken in Spring 2002. The actual intervention duration was from late November 2000 to May 2002, spanning a period of approximately 1 1/2 school years. Written parental consent and student assent were obtained before participation in the measurements and for publication of study results. The study was approved by the Human Subjects Review Committees at the University of Texas-Houston, School of Public Health, Baylor College of Medicine, and school district research committees.

### The IMPACT Physical Activity Intervention

The IMPACT intervention was based on the theoretical foundations of the Social Cognitive Theory and the Trans-Theoretical Model. These theories integrate determinants of behavior (such as outcome expectations, self-efficacy, behavioral capability, and environment) with methods of behavior change. In accordance with the premises of these theories, the IMPACT intervention sought to affect behavior change through promoting active learning in classrooms, as well as through environmental reinforcement. To this end, the intervention consisted of three major components: a health curriculum for grades 6 and 7 which included classroom lessons and behavioral journalism, a physical education program, and a school food service component that emphasized calcium rich food choices. The use of peer-based behavioral journalism involved the use of media such as a school-based newsletter with role model stories to increase adoption of desired behaviors. The over-arching goal of the physical activity component of the IMPACT trial was to improve bone health in the study sample by increasing overall levels of physical activity, specifically focusing on increasing weight bearing physical activity. To this end, the intervention employed a 6^th ^grade health curriculum which included 16 sessions that were implemented during physical education classes (3 times/week). The lessons in this curriculum were designed to promote increased consumption of calcium-rich foods and increased activity, specifically weight-bearing physical activities, while participating in behaviorally-based and active lessons adapted to the physical education environment. During 7^th ^grade, a series of science-based lessons were administered during science classes. The physical education component of the program known as *IMPACTivities*, was implemented in the 6^th ^and 7^th ^grades during physical education (PE) and athletics classes. The PE classes focused on an initial 10 minute warm-up (range: 5–15 minutes), which consisted of high impact activities such as rope-jumping, circuit training and box-step activities. This time was included in the outcome measure for WBPA. The overall emphasis of the PE program was to increase the duration of WBPA as well as overall levels of MVPA.

### Program Implementation and Process Measures

Various measures were used to insure the proper implementation of the physical activity intervention at the school level. These measures included regular observation of physical education and health classes and teacher checklists for completion of designated lessons, as well as observation of specific lessons. During 7^th ^grade, the number of advisory periods and newsletters, as well as the number of science lessons taught were enumerated for each school.

### Exposure and Outcome(s) Assessment

The main exposure for the presented analysis was the IMPACT intervention. Main outcome variables related to activity included: 1) duration of WBPA, MVPA and VPA, 2) duration of sedentary activities before and after school on weekdays and on weekends, and 3) duration of physical activity outside of the school, i.e. before and after school on weekdays and on weekends. For each outcome of interest, baseline and follow up values were calculated and compared between participating intervention and control schools.

### Individual Level Physical Activity Measures

Physical and sedentary activity data were collected using multiple administrations of the Self-Administered Physical Activity Checklist (SAPAC), as well as with the Calcium, Osteoporosis and Physical Activity (COPA) questionnaire.

The SAPAC was the primary instrument to assess total activity, WBPA, MVPA, and VPA. The SAPAC collected data on the intensity, duration and types of physical activity and consisted of 25 physical activities with spaces for listing up to three "other" activities that may have not been included in the main activity list. The SAPAC also had an additional section for sedentary activities such as television and video watching at three time periods during the previous day, i.e. before, during and after school. The self-report version of the SAPAC was previously validated against heart rate monitors (r = 0.57, p = .0001), and interviewer-administered checklists, (r = 0.76, p = .0001) in 125 fifth-grade children from four regions of the United States [26]. To improve precision and reliability, the SAPAC was administered to study participants on three separate days, which included one weekend day and two random weekdays, and an average score was calculated for each participant.

Values for each category of physical activity (MVPA, WBPA, VPA, etc.) were calculated using the sum of the minutes of the activity corresponding to these intensity levels for each student. Sedentary activity levels were measured using three variables from the SAPAC: i) mean daily minutes of television-video viewing; ii) mean daily minutes of computer-video game playing; and iii) mean daily minutes of combined sedentary behavior (sum of television-video viewing and computer-video game playing minutes). Sedentary variable values represent mean values from the three SAPAC administrations for each student.

Prior to analysis, items on the SAPAC were coded to provide information on weight bearing physical activities and metabolic equivalent values. Classification of activities as WBPA or NWBPA (non-weight bearing physical activities) was obtained from previously cited literature on the subject [27,28]. SAPAC items were assigned a metabolic equivalent or MET value based on established guidelines [29]. The MET value provides an accurate estimate of the intensity of a physical activity and also provides a reliable measure of energy expenditure during an activity relative to energy expenditure at rest [29,30].

The COPA (Calcium, Osteoporosis and Physical Activity Questionnaire) was primarily used to gather data on psychosocial variables related to calcium intake, physical activity and osteoporosis. The COPA was specifically developed for IMPACT and the key constructs measured by this instrument included variables such as calcium knowledge; weight bearing exercise and osteoporosis knowledge; physical activity expectations, etc. The COPA was developed and adapted from previous instruments such as the School Based Nutrition Monitoring (SBNM) student questionnaire [31] and the Health Behavior Survey [32]. The COPA was evaluated for reproducibility using a sample of 93 sixth grade girls from the same sample with duplicate administration of the questionnaires 11 days apart. Reproducibility was evaluated using percent agreement or Spearman correlations. The correlations for physical activity items ranged from 0.42 to 0.67. An evaluation of the physical activity self-efficacy scale using the baseline IMPACT sample revealed a Cronbach's alpha value of 0.87.

Seven items on the COPA directly assessed student participation in physical activity and support from friends and family for the same. These items asked students to rate the following: i) how often during the past month their family did physical activity with them; ii) how often during the past month their family encouraged them to do physical activity; iii) how often during the past month their friends did physical activity with them outside of school; iv) how often during the past month their friends encouraged them to do physical activity; v) how safe it was for them to play outdoors in the neighborhood with their friends without adult supervision; vi) how many sports teams they were on during the past 12 months (not including PE classes); and vii) whether they were currently participating in any other organized physical activity such as martial arts, gymnastics or tennis. Answers to these questions from the COPA were used for data on covariates.

The instruments and details on their administration protocols may be viewed at the IMPACT project website at: 

### Data analysis

The main endpoints for this study were measures of physical activity (WBPA, MVPA, VPA, total of all activities, sedentary activities, before/after school activities, and weekend activities). The analysis for the above variables was performed using mixed model analysis of covariance (ANCOVA) on the calculated scores for each variable (pre- and post-intervention) to test for differences among groups using specific techniques applicable to the unique challenges associated with the analysis of group-randomized trials. The essence of the unique methodological issue that arises with a group randomized design is that the condition (intervention/control) is assigned at the group (school) level, whereas the data is gathered at the member (student) level. Since students in a cluster (in this case, the school) are more likely to be similar to each other than to students in other schools, an adjustment is required to account for the clustering of values within schools. This is due to the fact that the *within-schools *correlation in the data, indexed by the intra-class correlation coefficient (ICC) adds an additional component of variance to the variability of the means in the intervention group, over and above the variance attributable either to the individual units (students) or to the intervention itself. It is essential to account for this *within-schools *correlation component during analysis in order to prevent inflated estimates of statistically significant differences between groups (represented by exaggeratedly lower p-values and lower estimates of standard error) than those that actually exist [33].

A pretest-posttest control group design analysis using the General Linear Mixed Model was utilized for the data obtained for this study. The variables were analyzed with the intervention arm (intervention/control) as the independent variable, and the specific physical activity/sedentary activity endpoints as the dependent variables. The analysis examined change in the dependent variables over time adjusting for baseline differences. The covariates examined were specific to the relevant response variable, but included ethnicity, body mass index, menarcheal status (yes/no), participation in PE/sports teams, neighborhood safety, and familial/friend support. Posttest data were analyzed with regression adjustment for covariates measured at baseline, thereby including time-related information without modeling time explicitly in the analysis [33]. This option was particularly suitable to our study as data was considered from only two time intervals, thus making it possible for time to be reflected only indirectly in the analysis of posttest data that made a regression adjustment for baseline values [34]. Time and treatment condition were modeled as fixed effects, with the individual student modeled as a random effect, and the school unit modeled as a nested random effect. This model thus accounted for variation effects attributable to the schools, to residual error, and to the heterogeneity among the school-specific slopes.

All statistical analyses were conducted using STATA version 9.0 program for longitudinal and mixed modeling, a mixed model regression program specifically suited for analysis of data from group-randomized trials [35].

## Results

Twelve schools had initially been recruited for the study and they remained in the study throughout the 1 1/2 year intervention period; however, results from process evaluation indicated that one of the intervention schools did not complete any of the IMPACT curriculum lessons during the first year, and no health education lessons were observed during process evaluation and quality control visits. Due to the absence of documentation that this school implemented the curriculum and the physical education components of the intervention during Year 1, the final results for key variables presented here exclude this school. The final sample size analyzed with 11 schools (5 intervention and 6 control schools) included 606 girls at follow-up. Thus, the current paper does not present an intent-to-treat analysis, but presents critical outcomes for the schools that actually participated in the IMPACT intervention.

### Description of Study Participants

Study participants were mostly white (72%) with 12% Hispanic and 5% African-American (Table 1). Mean age at baseline was 11.6 years (± 0.4 years) compared to 13.2 years (± 0.4 years) at the conclusion of the study. Most of the girls at baseline were pre-menstrual (20% menstruating at baseline) relative to 67% menstruating at the follow-up phase of the study.

**Table 1:** Participant Demographics for IMPACT at Baseline and Follow-up Measurement Periods, 2000–2002.

**CHARACTERISTIC**	**BASELINE (Fall 2000)**		**FOLLOW-UP (Spring 2002)**	
	**Number**	**Percent**	**Number**	**Percent**

**TOTAL STUDENTS**	718		606	
**Control**	371	52%	315	52%
**Intervention**	347	48%	291	48%
**ETHNICITY**				
**Non-Hispanic White**	515	72%	436	72%
**Hispanic**	83	12%	73	12%
**African-American**	39	5%	27	5%
**Other**	80	11%	69	11%
**ONSET OF MENSES**	140	20%	407	67%
	**Mean**	**SD**	**Mean**	**SD**
**AGE IN YEARS**	11.6	± 0.4	13.2	± 0.4

### Physical Activities

In each category of the physical activity variables (WBPA, MVPA, VPA, MET-weighted WBPA and MVPA), increases in activity levels were observed in the expected direction in intervention schools relative to the control schools, but results were only significant for VPA. Relative to the students in the control schools, students in the intervention schools engaged in more daily minutes of WBPA (difference = 5.12 minutes, 95% CI = 4.82–5.42, p = 0.33) at follow up compared to baseline (Table 2). Although not statistically significant, this represents an increase of approximately 6.0% in daily WBPA from baseline for students in the intervention schools, while WBPA for students in the control schools increased by 1.6%. Relative to students in the control schools, students in the intervention schools had higher overall total daily minutes of physical activity and daily MVPA minutes, although neither change was statistically significant. In contrast, the total daily minutes of vigorous physical activity (VPA) were significantly higher at follow-up for girls enrolled in intervention schools (difference = 6 minutes, 95% CI = 5.82–6.18, p = 0.05) compared to girls in control schools. This represents an increase of 45.4% in VPA minutes from baseline for students in intervention schools, while VPA minutes at follow-up relative to baseline *decreased *for students in control schools by 4.1%. There were no statistically significant changes in MET weighted minutes for both WBPA and MVPA (Table 2).

**Table 2:** Intervention Effects as Measured in 2002, Relative to Baseline (2000) for Selected Key Physical Activity Endpoints for the IMPACT Cohort (N = 11 schools)

**Variable**	**Baseline Mean (SD)**	**Follow-Up Mean^a ^(SE)**	**Difference^b^**	**95% CI**	**P-Value**
WBPA (Daily Weight Bearing Minutes)					
C	85.66 (58.13)	87.05 (3.74)	5.12 ↑	4.82 – 5.42	0.33
I	87.11 (51.92)	92.17 (3.64)			
WBPA (Number of Daily Weight Bearing Activities)					
C	3.12 (1.95)	3.18 (0.14)	0.25 ↑	0.23 – 0.27	0.23
I	3.19 (1.39)	3.43 (0.14)			
Total Daily Minutes of Activity					
C	102.63 (65.32)	104.30 (4.24)	6.15 ↑	5.81 – 6.49	0.30
I	103.55 (61.82)	110.45 (4.13)			
MVPA (Daily Moderate to Vigorous Physical Activity Minutes)					
C	64.63 (53.93)	63.63 (3.63)	7.05 ↑	6.75 – 7.35	0.16
I	64.85 (47.23)	70.68 (3.53)			
Total Daily Minutes of Vigorous Activity					
C	14.96 (28.39)	14.35 (2.24)	6.00↑	5.82 – 6.18	0.05*
I	14.00 (29.33)	20.35 (2.18)			
Daily Total Weight Bearing Adjusted MET Value (mins)					
C	400.01 (284.17)	415.53 (20.29)	43.27↑	41.62 – 44.92	0.13
I	411.68 (303.51)	458.80 (19.71)			
Daily Total Moderate to Vig. Activity Adjusted MET Value (mins)					
C	380.53 (330.81)	383.07 (22.90)	40.74 ↑	38.88 – 42.60	0.20
I	382.25 (307.92)	423.81 (22.24)			

^a ^Adjusted follow-up value in a mixed model with individual student modeled as random effect and school as nested random effect; time and condition as fixed effects; covariates include ethnicity, menstrual status, body mass index, participation in 7^th ^grade PE/athletics.

^b ^Adjusted difference at follow-up between intervention (I) and control (C); adjusted for baseline scores of variable, ethnicity, menstrual status, body mass index, participation in 7^th ^Grade PE/athletics.

### Before/After School Activity and Weekend Minutes

Students in intervention schools also spent approximately 9 more minutes per day (95% CI = 8.69–9.21, p = 0.04) than those in control schools in daily after school physical activity at follow-up, suggesting a statistically significant difference in the follow-up means of the two groups (Table 3). This represents an increase of 13.3% in daily after school activity minutes from baseline for students in intervention schools, while those in control schools increased by 1.7% for the same variable. Students in intervention schools spent significantly higher number of minutes in daily weekend physical activity (difference = 19 minutes per day, 95% CI = 18.40–19.60, p = 0.05) relative to those in control schools at follow up. There was no statistically significant difference in the follow-up means of the intervention and control groups with respect to daily before school activity minutes.

**Table 3:** Baseline Values and Intervention Effects for Before/After School Activities, Weekend Activities, and Sedentary Activities as measured by the SAPAC for the IMPACT Cohort (N = 11 schools)

**Variable**	**Baseline Mean (SD)**	**Follow-Up Mean^a ^(SE)**	**Difference^b^**	**95% CI**	**P-Value**
**Before/After School Activity and Daily Weekend Activity**					
Daily Before School Activity Minutes					
C	7.50 (11.05)	6.54 (0.80)	1.83 ↑	1.76 – 1.90	0.10
I	7.00 (11.87)	8.37 (0.78)			
Daily After School Activity Minutes					
C	60.83 (37.92)	61.89 (3.23)	8.95* ↑	8.69 – 9.21	0.04*
I	62.50 (40.60)	70.84 (3.15)			
Daily Weekend Activity Minutes					
C	102.00 (77.87)	107.00 (7.37)	19.00*↑	18.40 – 19.60	0.05*
I	102.50 (76.50)	126.00 (6.93)			
					
**Sedentary Activities**					
Daily TV and Video Minutes					
C	90.62 (83.37)	106.81 (4.59)	12.11*↓	11.74 – 12.48	0.05*
I	113.72 (89.27)	94.70 (4.40)			
Daily Computer/Video Games Minutes					
C	23.16 (38.31)	44.36 (3.29)	6.32 ↓	6.05 – 6.60	0.16
I	23.49 (36.34)	38.04 (3.12)			
Total Daily Sedentary Activity Minutes					
C	113.78 (97.77)	151.91 (5.95)	16.99*↓	16.49 – 17.50	0.04*
I	137.22 (99.23)	134.92 (5.68)			

^a ^Adjusted follow-up value in a mixed model with individual student modeled as random effect and school as nested random effect; time and condition as fixed effects; covariates include ethnicity, menstrual status, body mass index, participation in 7^th ^grade PE/athletics, neighborhood safety, family and friend support for physical activity.

^b ^Adjusted difference at follow-up between intervention (I) and control (C); adjusted for baseline scores of variable, ethnicity, menstrual status, body mass index, participation in 7^th^. Grade PE/athletics, neighborhood safety, family and friend support for physical activity.

### Sedentary Activity Minutes

Relative to girls in the control schools, daily TV and video minutes were *lower *for students in the intervention schools at follow-up (↓12.11 minutes, 95% CI = 11.74–12.48, p = 0.05) suggesting a decrease in TV/video watching from baseline for students in intervention schools of 16.7%, while TV/video watching in students in the control schools *increased *by 17.9% (Table 3). Total daily minutes of sedentary activity were significantly lower for students in intervention schools relative to those in control schools at follow-up (↓17 minutes, 95% CI = 16.49–17.50, p = 0.04). This represents a decrease in sedentary activity minutes from baseline for students in intervention schools of 1.7%, while sedentary activity minutes in students in control schools *increased *by 33.5%.

## Discussion

Results from our study indicate that for each of the categories of key physical activity variables examined (*weight-bearing (WBPA), aerobic (MVPA and VPA), and MET-weighted WBPA and MVPA*), intervention schools had higher follow-up means relative to control schools, but onlychanges in VPA were statistically significant. With respect to the each of the categories of the remaining endpoints examined (*before/after school activities, weekend activities, and sedentary activities*), results were statistically significant for: daily after school activity minutes, daily weekend activity minutes, daily TV and video minutes, and total daily sedentary activity minutes.

The IMPACT major results paper examined data obtained from the IMPACT study using an intent to treat analysis which included all 12 schools in the study's sample [36]. However, this paper included only three physical activity variables that were the main hypotheses of that study: mean daily minutes of activity, mean daily minutes of VPA, and mean daily minutes of WBPA. No other variables from the SAPAC physical activity instrument were included in the major results paper. In addition, data for sedentary activities were also not included in the IMPACT major results paper [36]. Using hierarchical modeling and the same covariates as in our study, it was found that no significant changes were obtained in the stated three physical activity variables in the intent to treat analysis [36]. In comparison to those results, data from the current study show that significant gains were obtained in one of the main physical activity variables (mean daily minutes of VPA) in the schools that actually participated in the intervention.

The majority of other intervention studies [14-25] that have focused on promoting bone accretion have not been formally evaluated with respect to the actual effectiveness of their physical activity programs in terms of measurable differences in the duration of WBPA or MVPA pre- and post-intervention. Recent intervention studies (both school and clinic/HMO-based) have reported mixed results with respect to net gains obtained in WBPA or MVPA through interventions targeted at middle-school age or older adolescents [16,18,19,37,38]. Our results corroborate with those obtained from French et al. and Ievers-Landis et al. [18,19]. Although both the French et al. and Ievers-Landis et al. studies had relatively small sample sizes utilizing Girls Scout troop populations, both studies reported no significant intervention effects for increases in WBPA. These studies attributed the lack of a significant effect in WBPA due to greater focus on improvement in behavioral variables rather than a structured and supervised PA program emphasizing standardized frequency, duration and type of PA [18], as well as due to limited participation [19]. Students in the IMPACT intervention schools received a 10–15 minutes initial warm-up of high impact exercises plus their regular PE class with the objective being to achieve an increased duration of WBPA in this group relative to the control group. Although no upper limit has been established with respect to WBPA activity levels, it is generally understood that increased duration or intensity of weight bearing activity translates to improved bone health [11,12]. Although the IMPACT intervention group had somewhat higher WBPA levels than the control group, this gain was not significant statistically, and more importantly, it was not sufficient with respect to expecting lasting improvements in bone health in the intervention group. Results from our study as well as from studies cited above suggest that more structured and closely supervised programs aimed at increasing both the frequency and the duration of weight bearing activities may yield higher gains in future interventions for this variable.

Results with respect to MVPA and daily total minutes of VPA suggest improvements in physical activity levels in the intervention schools relative to control schools. For each of these variables, the net percentage increase from baseline values for intervention schools is particularly noteworthy as duration of both MVPA and VPA *decreased *in the control schools: for MVPA, intervention schools increased by 9.0% from baseline values while control schools *decreased *by 1.5%; for VPA, intervention schools increased by 45.4% from baseline values while control schools *decreased *by 4.1%. Results for MVPA are supported by those obtained in a recent study by McKenzie et al. involving a 2 year middle school intervention during PE classes, in which MVPA in PE classes was reported to have increased by 3 minutes per lesson (p = 0.02) [16]. Results from the Child and Adolescent Trial for Cardiovascular Health (CATCH) indicated substantially greater MVPA during lessons for students in intervention schools relative to those in control schools (51.9% vs. 42.3% of lesson time, p = 0.002). In addition, total PE minutes per week at follow up were higher in students from CATCH intervention schools by 4.4 minutes (p = 0.19) [17]. It must be noted, however, that relative to our study, both of these studies [16,17] had substantially larger sample sizes allowing for significantly more statistical power to detect differences between comparison groups.

Results obtained from our analysis for daily total MET-weighted minutes for WBPA and MVPA similarly show improvement in values from baseline for the students in the intervention schools relative to students in the control schools. Both WBPA and MVPA METS were higher for intervention schools at follow-up (43.27 minutes, p = 0.13 and 40.74 minutes, p = 0.20, respectively). The difference between intervention and control groups in general MET weighted physical activity minutes reported by the CATCH study was 43 minutes, p = 0.22 [17]. To put our results in perspective, it must be kept in mind that the CATCH analysis for this variable did not involve hierarchical modeling and represents individual level effects which generally tend to overestimate results.

Studies that address reduction in television viewing or computer/video game use are limited and generally linked with programs designed for obesity reduction in children [39-41]. We can thus only compare our findings to these studies, the majority of which were interventions specifically targeted at reducing TV viewing. Results from most of these studies indicate substantial reduction in TV viewing time. Dennison et al. reported an adjusted difference between groups of -4.7 hours/week, p = 0.02 [39]; Robinson et al. reported an adjusted difference between groups of -4.96 hours/week, p = 0.007 [40]; while Gortmaker et al. reported a difference between groups of 0.55 hours/day, p = 0.05 [41]. Although the IMPACT intervention was not specifically targeted at reducing TV viewing or computer use, one of the more striking common elements of the above-mentioned studies and IMPACT appears to be that students were provided with tangible ideas and attractive alternatives to sedentary activity/TV watching (e.g. specific suggestions for being active in the evenings and on weekends; specific venues to enjoy physical activity, etc.). We believe that the reduction in sedentary behaviors observed in this study may be due, in large part, to the provision of concrete suggestions, ideas and ways for students to keep active. The participation in active rather than sedentary behaviors was further positively reinforced at school via the IMPACT curriculum lessons.

We do not have suitable comparison studies with respect to variables that represent intervention-influenced behavior modification outside of the school (before/after school activity minutes, and weekend activity minutes). The majority of previous research has not reported the effects of school based interventions on behavior modification *outside *of the school. Thus, our study is one of the first to report how a school based intervention can influence levels of sedentary and physical activity behaviors *both in and out of *school. We believe that one of the reasons for the positive findings with respect to after-school and weekend physical activity levels may be that the intervention strongly encouraged students to engage in outdoor activity. Students were provided ideas and suggestions for staying active on both evenings and weekends. Furthermore, since the IMPACT study population was primarily from white, suburban neighborhoods, neighborhood-safety was not an issue for most students. This factor may have played an important role in the maintenance of activity levels outside of the school. Our analysis clearly indicated that factors such as neighborhood safety, friend/family support, and friend/family involvement (data not shown) in physical activity were critically important covariates in determining accurate levels of activity outside of the school and on weekends. Findings with respect to social support are largely consistent with those obtained by Springer et al. in a recent study [42].

### Strengths and Limitations

The main strengths of our study include its strong study design, high quality measurement of outcomes, and high reliability and validity of measurement instruments. In particular, one of the strengths of the study is that the physical activity data collection instrument, the SAPAC was administered three times within a single week at baseline (two weekdays, and one weekend day), reducing variability of this measure from 40 to 25% (data not shown). The majority of PA variables were calculated from the averages obtained from the reported PA levels for three days. This provided us with a more reliable estimate of the subjects' daily activity patterns during the course of both weekdays and weekends.

One of the potential weaknesses of our study includes the evaluation of data at two distinct time periods only, i.e. at baseline and follow-up. Data obtained through process evaluation and for other measures (e.g. the dietary intakes) suggest that the intervention was most effectively implemented in the first year (6^th ^grade). For several key behavioral variables (e.g. calcium intake), optimal (peak) effects were obtained at the interim measurement phase of the intervention, followed by a moderate decline in the variable values at the final measurement phase of the study. While data were collected on nutrition-related variables at the interim phase of the study, data were not collected for the physical activity variables at this stage of the study, thus precluding the possibility of analyzing trends in physical activity patterns over the entire duration of the intervention. In light of the study implementation being close to optimal in the first year of the intervention, it is conceivable that more significant effects in WBPA and MVPA may have been obtained at the interim phase of the study.

Another limitation of the study is the relatively few number of groups randomized to treatment conditions. This feature of our study is quite typical of group-randomized trials due to the costs involved in recruiting and administering the intervention to the groups. Although our study utilized a strong analytical approach and had sufficient power to detect differences between the groups, a higher number of groups would yield greater statistical power and accuracy in determining intervention effects.

Additional potential study limitations include reliance on self-reported measures of physical activity (generally leading to an over-reporting of PA levels) and limited information on additional possible covariates such as parental involvement. Data from other PA studies suggest that parental involvement in school or community based interventions may improve compliance and participation in intervention activities [19].

## Conclusion and recommendations

Results from our study indicate that a school based health and physical activity intervention can have a positive influence upon increasing physical activity levels as well as decreasing sedentary activity both in and outside of the school environment. Although we did not observe statistically significant changes in WBPA and MVPA, the intervention resulted in an overall increase in all physical activity scores for the participating intervention schools relative to the control schools, and in a statistically significant reduction in the duration of student daily TV/Video watching and daily sedentary activity minutes. Our results thus indicate that with careful implementation, emphasis on staff development and regular follow-up throughout the duration of the program, changes in physical activity behaviors can be achieved. However, significant and lasting improvements may depend upon more structured and standardized programs, especially with regard to WBPA. Future research must focus specifically on the development of controlled studies that examine more specific and measurable physical activity dosages (both WBPA and MVPA); examination of results at more frequent measurement intervals; and finally, strategies that insure that effects obtained are of a lasting nature. In addition to the recommendations cited previously, these strategies may include a higher parental involvement component, as well as longer study duration to instill changes in exercise habits that may be incorporated permanently into the student's lifestyle.

## Competing interests

The authors declare that they have no competing interests.

## Authors' contributions

DJ: Study conception and design; statistical analysis and interpretation of data; primary role in drafting of the manuscript; and critical review of the manuscript. DH: Obtaining funding for the study; acquisition of data; study conception and design; administrative, technical and material support; and critical review of the manuscript. SK: Study conception and design; administrative, technical and material support; review of manuscript. AH: Study conception and design, review of manuscript. SS: Assistance with analysis of data; review of manuscript. All authors read and approved the final manuscript.

## Availability and requirements


















